# Uncovering the impact of AM fungi on wheat nutrient uptake, ion homeostasis, oxidative stress, and antioxidant defense under salinity stress

**DOI:** 10.1038/s41598-023-35148-x

**Published:** 2023-05-22

**Authors:** Shoucheng Huang, Sidra Gill, Musarrat Ramzan, Muhammad Zaheer Ahmad, Subhan Danish, Ping Huang, Sami Al Obaid, Sulaiman Ali Alharbi

**Affiliations:** 1 College of Life and Health Science, Anhui Science and Technology University, Fengyang, 233100 China; 2 Department of Botany, Faculty of Chemical and Biological Sciences, The Islamia University of Bahawalpur, Bahawalpur, Pakistan; 3 Dr. M. Ajmal Khan, Insititute of Sustainable Halophytes Utilization, University of Karachi, Karachi, Pakistan; 4 Department of Soil Science, Faculty of Agricultural Sciences and Technology, Bahauddin Zakariya University, Multan, Punjab Pakistan; 5 College of Chemistry and Materials Engineering, Anhui Science and Technology University, Bengbu, 233000 China; 6 Department of Botany and Microbiology, College of Science, King Saud University, PO Box -2455, Riyadh, 11451 Saudi Arabia

**Keywords:** Salt, Plant stress responses, Plant sciences, Plant symbiosis, Arbuscular mycorrhiza

## Abstract

The growth of wheat (*Triticum aestivum*) is constrained by soil salinity, although some fungal species have been shown to enhance production in saline environments. The yield of grain crops is affected by salt stress, and this study aimed to investigate how arbuscular mycorrhizal fungus (AMF) mitigates salt stress. An experiment was conducted to assess the impact of AMF on wheat growth and yield in conditions of 200 mM salt stress. Wheat seeds were coated with AMF at a rate of 0.1 g (10^8^ spores) during sowing. The results of the experiment demonstrated that AMF inoculation led to a significant improvement in the growth attributes of wheat, including root and shoot length, fresh and dry weight of root and shoot. Furthermore, a significant increase in chlorophyll a, b, total, and carotenoids was observed in the S2 AMF treatment, validating the effectiveness of AMF in enhancing wheat growth under salt stress conditions. Additionally, AMF application reduced the negative effects of salinity stress by increasing the uptake of micronutrients such as Zn, Fe, Cu, and Mn while regulating the uptake of Na (decrease) and K (increase) under salinity stress. In conclusion, this study confirms that AMF is a successful strategy for reducing the negative effects of salt stress on wheat growth and yield. However, further investigations are recommended at the field level under different cereal crops to establish AMF as a more effective amendment for the alleviation of salinity stress in wheat.

## Introduction

**Figure 1:**
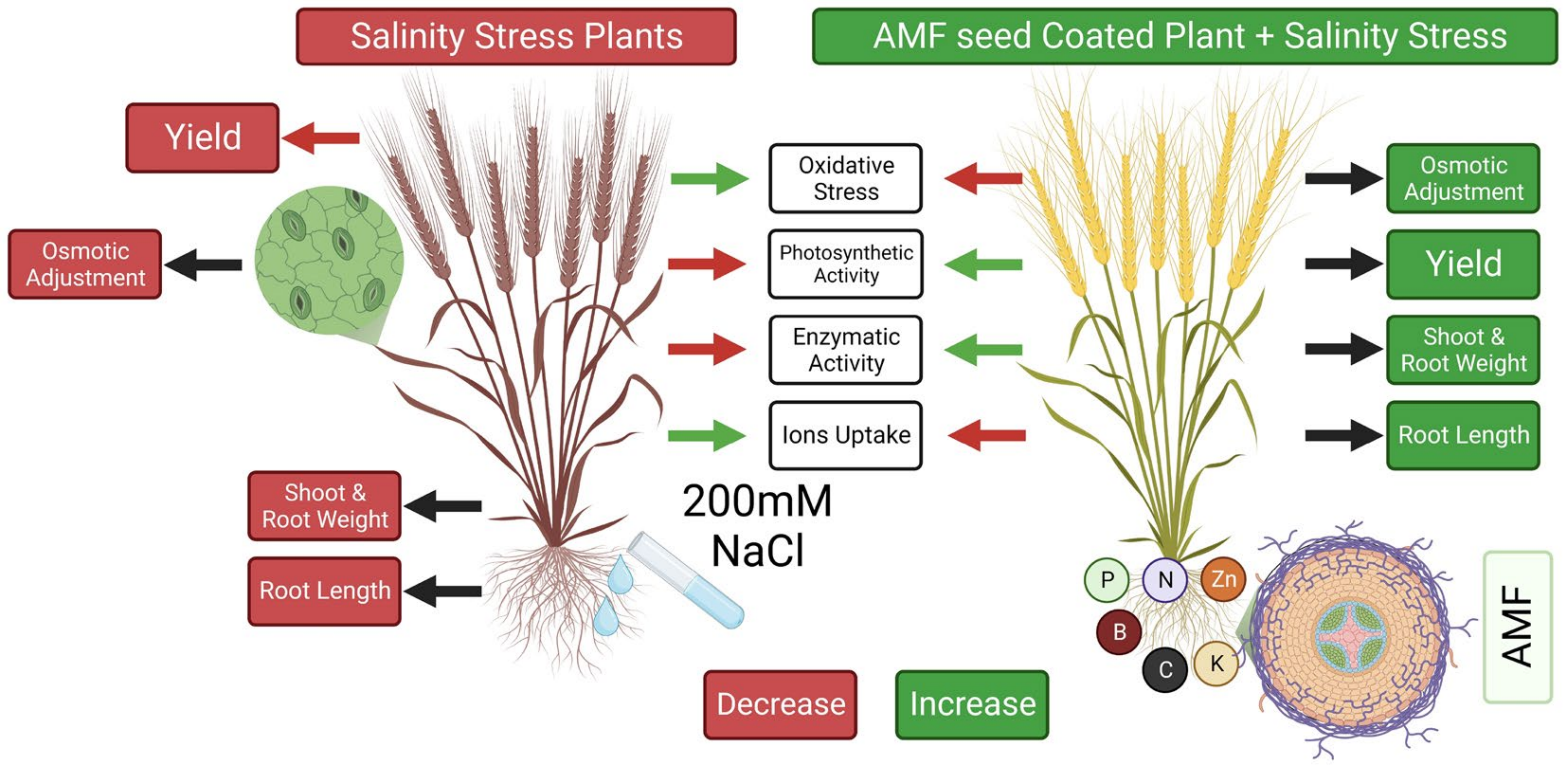
Graphical abstract showing the major impacts od treatments on wheat in current study (self generated by using software Biorender).

In arid and semi-arid areas of the world, salt stress has become a challenge to agricultural output^1^. A single abiotic stress on plant progression as well as development is one of the abiotic stresses on plant growth and development that eventually causes a drop in production^2^. Salt stress affects 20% of the biosphere's arable land, worsening because of human activities and global warming^3^. Salinity is a type of environmental stress that can contribute to around 50% of output losses^4,5^. Salt stress impairs plant growth as well as production by putting osmotic stress, which causes ion poisonousness and nutritional disparity in plants^2,6^. Plants under salt stress experience physiological, biochemical, morphological, as well as molecular alterations^2,7,8^. Salinity also alters the ultrastructure of cells, hinders photosynthesis, damages membrane structures, increases the production of responsive oxygen agents, and inhibits enzymatic activity, all of which harm crop development and yield^9,10^ (Fig. 1).

Soil salt has a harmful effect on various wheat plant morphological characteristics, together with seedling germination, plant length, chlorophyll content, shoot, as well as root length, leaf area, the number of roots, leaves, root/shoot ratio, and fresh as well as dry weight. While^9,11^ institute that plumule length was more responsive for the period of primary growth stages, salt stress resulted in the early maturity of wheat, which lowered the plant height as well as leaf area. According to a recent study, the changed leaf and stem anatomical characteristics of several wheat genotypes are crucial for adaptation to salt stress^12^. The physical variations that occur for the duration of leaf senescence due to various stressors have mostly been studied concerning the damage of photosynthetic pigments, protein breakdown, and re-absorption of mineral nutrients^13,14^. Based on^15^, variations in physiology and metabolism are specific to each stage and can impact the ultimate yield of the process. They asserted that saline lowers grain output at several phases, including anthesis, mid-grain filling, as well as early boosting. Salt stress lowered wheat production potential by accelerating the shoot apex's growth, lowering the total of spikelet primordia, as well as inducing premature terminal spikelet stage as well as anthesis^16,17^.

Soil microbes like AMF provide an essential association amongst plants and the soil's mineral nutrients. The soil microbiota, often called “agroecosystem engineers,” is vital for crop productivity, soil fertility, ecosystem resilience, yield, and quality. AMF is a vital constituent of this micro-biota. They are, therefore, essential for agriculture^18^. AMF is essential to agriculture since it might reduce the need for synthetic fertilizers^19,20^. AMF establish symbiotic associations with plant roots and also aid in delivering essential nutrients to hosts or occupied plants, improving their development, photosynthesis, as well as crop yields. It increases the roots' access to and exposure to a larger region of the soil surface and develops a hyphal network inside the roots^21^. AMF assists in refining soil erection, quality, also structure by accelerating the breakdown of soil organic matter^22^.

The ability of AMF to assist atmospheric CO_2_ fixation in host plants is well established^23^. Increased photosynthetic efficacy was detected in AMF recipient crops in salt stress^24^. It has been demonstrated that AMF symbiosis is advantageous when it increases photosynthetic rate, leaf water relations in saline settings, and stomatal conductance. Na transport was reduced in plants infected with AMF under saline conditions, although N and Mg absorption and chlorophyll content showed improvements^25^. AMF inoculation could significantly boost the number of photosynthetic pigments present, slow down Chl's degradation beneath salt stress, also improve photophosphorylase action^21^. Mycorrhizal symbiosis lessens the detrimental effects of salinity on plant productivity by several appliances, including shielding roots from soil-borne pathogens, refining antioxidant enzyme activity, sustaining membrane permeability, activating plant growth regulators, boosting nutrient absorption, sustaining K^+^/Na^+^ ratio, as well as inducing biochemical changes (prolactin accumulation)^26,27^.

Wheat is a portion of basic food for 35% of the world's inhabitants. (Ministry of Food^28,29^. Wheat is a significant source of carbohydrates (55%) and a crop that supplies 20% of the world's food needs^30^. According to estimates, 696 million tons of wheat were produced between 2011 and 2012. It is the most significant crop in Pakistan, accounting for over 40% of all farmed land ^29^. Pakistan has the world's largest per-capita consumption of wheat, which is typically estimated to be around 124 kg per year^29,31^. Although wheat is one of the salinity-tolerant cereal crops, its production is decreased by salt attention above 100 mM NaCl^32,33^. Pakistan is one of the least developed nations, so it's crucial to use an efficient and long-lasting soil amendment to reduce production losses in crops growing under stress^29,34^. To comprehend salt tolerance mechanisms in AM plants, this study aimed to explore the impact of arbuscular mycorrhizal fungus on wheat plant development, chlorophyll fluorescence properties, antioxidant enzyme activity, photosynthetic pigments, mineral uptake, and yield under saline stress.

## Materials and methods

### Soil analysis

Three soil samples were taken, air-dried, then correctly combined before and after harvesting for a soil analysis to create a representative composite soil sample. The Kjeldahl method^35^ and the Olsen method^36^ measured N (0.002%) and available phosphorus (7.17 µg/g), respectively. The extraction procedure using ammonium acetate was used to assess the exchangeable K (85 µg/g) ^35^. However the soil organic matter was 0.35%^37^.

### Plant nutrient uptake analysis

The nutrient uptake of 12-week-old wheat plants was observed by analyzing dried-up as well as ground samples that were digested with H_2_SO_4_–H_2_O_2_ at temperatures between 260 and 270 °C. The Nitrogen content was measured using an Auto-analyzer 3 digital colorimeter (AA3, Bran + Luebbe, Germany, Hamburg), and the Potassium content was determined with flame photometry (Shanghai Precision Scientific Instrument, FP6400, China, Shanghai)^38^. The P concentration was assessed after digestion in nitric-perchloric acid with the Vanado-molybdophosphoric colorimetric technique. Each mineral's 10–100 g/mL standard curve was a reference.

### Experimental design and treatments

The* Triticum aestivum* (wheat) AAS-2011 variety was chosen for the experimentation. In the greenhouse experiment, selected healthy seeds were utilized. The seeds were carefully rinsed in distilled water after being surface sterilized for 5 min with a 0.1% mercuric chloride solution^39^. During the 2021–2022 wheat growing season, a pot experiment was conducted at the botany department at the Islamia University of Bahawalpur. The soil for the greenhouse experiment was gathered from the neighborhood nursery in Bahawalpur. The soil sciences division of Bahuddin Zakaria University in Multan, Pakistan, gave the AMF strain (*Glomus* spp.). AMF was applied to seeds at a rate of 0.1 g (10^8^ spores) when wheat was sown. 20 cm in diameter, as well as 20 cm shallow plastic pots filled with 6.0 kg of soil, were used for the seeding. Pots were divided into 4 groups: salt-treated soil, AMF-treated soil, soil not treated with AMF and NaCl as a control, and soil treated with salt and AMF. In this study, the concentration of NaCl solution in the pots was increased gradually from 50 to 200 mM every 24 h^40^. This gradual increase in concentration was done to minimize osmotic shock and root damage, and to allow the plants to acclimate to the salt stress gradually. The maximum concentration of 200 mM was chosen as the acceptable treatment threshold to induce salt stress in the plants for the experiment^41^. By testing the salinity with an EC meter at regular intervals, the appropriate salt levels and equal quantities were kept up until the plants were harvested^42^. After harvesting, cut the roots and shoots into pieces. The plant roots were washed with distilled water to remove any dust. Plant and soil samples were collected also dried in an oven for 48 h at 100 °C. The experiment design used was Completely Randomized Design (CRD). For each treatment, three replicates were employed. Each container held three plants. Greenhouse with a mean temperature of 30 °C, a photoperiod of 16 h of sunshine and 8 h of the night, 80% relative humidity, the experiment was carried out. Treatment information is included in Table 1.

**Table 1:** Treatments name and abbeviations.

Acronyms	Treatments
CK	Control
AMF	AMF inoculation alone
S2	200 mM NaCl alone
S2 + AMF	200 mM NaCl + AMF

### Measurement of morphological parameters

Plants from each treatment were uprooted at the end of the experiment. All the plants' roots and shoots were divided, and they were all washed separately. After washing, various morphological data were calculated, including the sum of leaves per plant, spikelets per plant, and tillers per plant. The shoot, spike, and root were measured in centimeters using the tape meter rule, and their fresh also dry weights were calculated in grams using an electronic balance. The dry mass of the shoots, spikes, plus roots of the harvested plants was determined after 48 h of storage at 70 °C in a dry oven.

### Estimation of chlorophyll contents

Wheat plants that were 12 weeks old had fresh leaves tested for chlorophyll analysis^43,44^. The leaves were carefully divided into tiny pieces (about 0.1 g), ground to a powder in 10 mL of 80% acetone also centrifuged for five minutes at 10,000 rpm. The process was reiterated after collecting the supernatant till the remainder had no color. The solution's absorbance was noted at 480, 645, and 663 nm. The blank solution contained 80 percent acetone. Using the method, the chlorophylls and carotenoids in the leaves were estimated to determine the photosynthetic pigments.

### Estimation of leaf relative water content

The technique was used to determine the relative water content (RWC)^45^. Fresh leaf samples weighing 100 mg, in the fully expanded condition, were put in Petri dishes having double-distilled water and left for four hours at room temperature. The samples were then taken out and dried off, and the turgor weight (TW) was also noted. The samples were then dried in an oven at 70 °C overnight; the dry weight (DW) was also noted. The relative water content was calculated using the method

$${\text{RWC}}\left( \% \right) = \left[ {\left( {{\text{FW}} - {\text{DW}}} \right)/\left( {{\text{TW}} - {\text{DW}}} \right)} \right] \times {1}00$$

where FW is the fresh weight of the tissue.

### Estimation of antioxidant enzyme

To extract protein, 12-week-old young wheat plants were harvested, and the leaves (1 g) were promptly lyophilized, freezing in liquid nitrogen, and then homogenized in 2 mL of potassium phosphate buffer (pH 7.8). The samples were centrifuged for 15 min at 4 °C and 12,000 rpm. Assays of enzymatic activity were conducted after the supernatants were collected into tubes and stored at 20 C.

To measure SOD activity, blue diformazan formation was suppressed by the influence of light and riboflavin/nitroblue tetrazolium (NBT). After that, a fluorescent lamp (75 W, 20 cm beyond the reaction fusion) was irradiated for 3 min while calculating the absorbance at 560 nm. SOD action is quantified in micrograms per milligram of protein, with one unit equaling a 50% reduction in the generation of blue diformazan^46^.

Using o-dianisidine as the substrate, the rate of rise in absorbance at 470 nm was used to determine POD activity^47^. As a min^−1^ mg^−1^ protein, the POD activity is expressed.

For the catalase (CAT) experiment, 2 mL of a 50 mM potassium phosphate buffer at pH 7 was used to mix 0.5 g of finely crushed dried oven leaf powder with 0.05% Triton X-100, 2% PVP, 1 mM EDTA, and 1 mM ascorbic acid. Rendering to ^48^, the mixture was centrifuged at 1000 rpm for 20 min at 4 °C, and the collected separation was used to gauge the CAT enzyme's action.

The activity of the enzyme ascorbate peroxidase (APX) was assessed by detecting a 1-min reduction in absorbance at 290 nm (Nakano and Asada, 1981). 0.15 mL of enzyme extract, 0.5 mM ASA, 0.1 mM H_2_O_2_, 0.1 mM EDTA, and 50 mM sodium phosphate buffer were used in the test mixture (pH 7.0).

### Estimation of electrolyte leakage in leaves

In a glass jar with leaf fragments, the old leaf was divided into 0.5 cm sections, wrapped in 7 cc of purified water, and then violently agitated at 120 °C for 30 min. The sample was autoclaved for 30 min at 120 °C, and then it was chilled to room temperature to acquire the analysis for initial leaf conductivity (EC-i) or final conductivity (EC-f)^20^,

$${\text{EL}} = \left( {{\text{EC}} - {\text{i}}} \right)/\left( {{\text{EC}} - {\text{f}}} \right) \times {1}00$$

### Estimation of hydrogen peroxide (H_2_O_2_)

A 0.25 g fresh leaf extract from the plants was standardized with 5% trichloroacetic acid (TCA, 3 mL), activated charcoal (0.1 g) at 0 °C, and then centrifuged at 12,000 rpm for 15 min. The separations were then diversified with 10 mM potassium phosphate buffer and 1 M potassium iodide in a pH 7.0 solution. The absorbance of the solution was calculated at 390 nm and used to determine the H_2_O_2_ concentration^49^.

### Malondialdehyde content measurement (MDA)

The malondialdehyde (MDA) concentration was observed by using the thiobarbituric acid (TBA) reaction^50^. 0.5 g of freshly washed leaves were mixed with 10 mL of 0.1% trichloroacetic acid and centrifuged at 4 °C for 15 min. 2 mL of the supernatant also 2 mL of thiobarbituric acid (0.67% w/v) solution were heated at 100 °C for 0.5 h. The supernatant was then transmitted to a cold bath and centrifuged at 10,000 g for 30 s at 4 °C. The absorbance was measured at 532 nm, and non-specific absorption was subtracted from the measurement at 600 nm. Malondialdehyde (MDA) absorption was calculated via MDA molar extinction coefficient.

### Total soluble protein content estimation

A standard curve made of different quantities of bovine serum albumin was used to measure the protein content (BSA). 1 mL of the leaf extract of a sample plant was added to a test tube, and 1 mL of pH 7.0 phosphate buffer was added. The reagent test tubes were left at room temperature for one minute. Folin-phenol reagent (0.5 mL) was added and then incubated for 30 min. The absorbance was detected at 620 nm with a spectrophotometer.

### Total soluble sugar estimation

The maximal soluble sugars were calculated using the Yemm and Willis method, which was created in 1954. 0.1 mL of the plant extract was added to 25 mL test tubes. To each tube, 6 mL of the anthrone reagent was added and heated in a boiling water bath for 10 min. The test tubes were cooled to room temperature for 10 min before incubating for 20 min. The absorbance was measured at 625 nm with a spectrophotometer.

### Ions estimation

Acid digestion of oven-dried (110 °C) leaf plus root samples followed by estimation of Na^+^, K^+^, NO_3_, and Cl using a flame photometer following Wolf's method from 1982 (Jenway Flame1104 A^51^. Photometer, Bibby Scientific Ltd-Stone-Staffs-St15 0SA–UK). The elemental (Mn, Fe, Cu, and Zn) concentration of the digested dried leaf powder was measured by atomic absorption spectrophotometer after adding 1 M hydrochloric acid to estimate Mn, Fe, Cu, and Zn.

### Statistical analysis

For this inquiry, the data were entered into the spreadsheet programs (Excel). The arithmetic mean or standard variation was determined. To analyze different treatments, a one-way ANOVA test was used. The treatments were compared using the two-way ANOVA and Tukey's Honest Significant Difference (HSD) Test. Applying OriginPro2021 software, correlation coefficient and principal component analysis were performed.

### Plant material collection and use permission

No permission is required for plant material. Seeds were pucrchased from local market.

### Ethics approval and consent to participate

We all declare that manuscript reporting studies do not involve any human participants, human data, or human tissue. So, it is not applicable.

### Complies with international, national and/or institutional guidelines

This study complies with relevant institutional, national, and international guidelines.

## Results

### Shoot length

**Figure 2:**
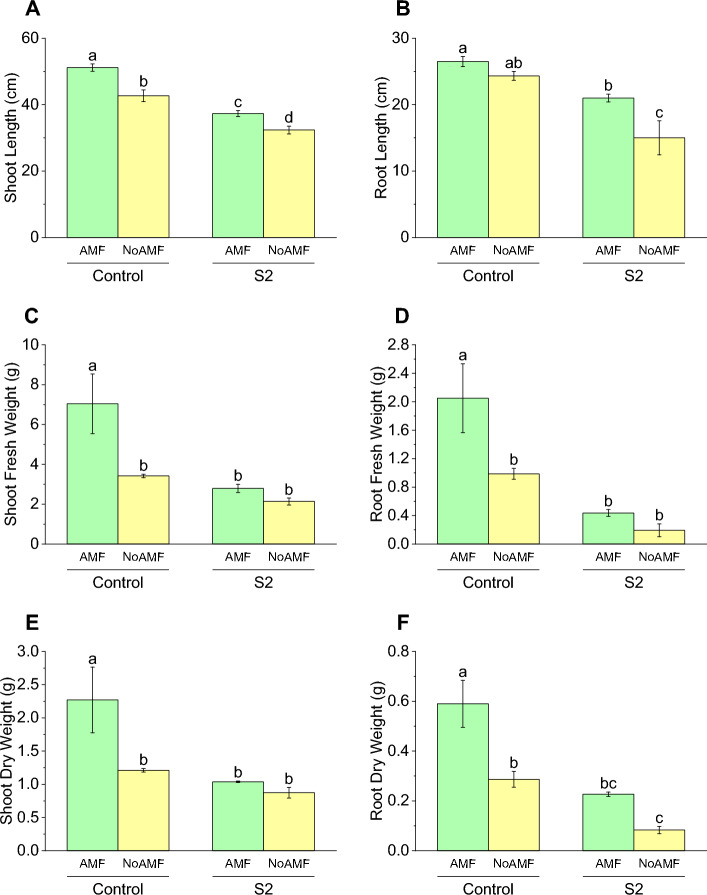
Effect of arbuscular mycorrhizal fungi (AMF) on shoot length (**A**), root length (**B**), shoot fresh weight (**C**), root fresh weight (**D**), shoot dry weight (**E**) and root dry weight (**F**) in wheat cultivated under normal and saline soil conditions (200 mM NaCl). The data reported are the mean values ± standard error of three independent replicates. Statistical analysis using Fisher's least significant difference (LSD) test showed that significant differences (*p* < 0.05) existed among the treatment groups. The use of different letters to label the means indicates the presence of significant differences between the groups.

The shoot length in the Control AMF treatment was significantly higher from the shoot length in the Control NoAMF. The shoot length in the S2 AMF treatment group was significantly lower than the shoot length in the Control No AMF treatment group. The shoot length in the S2 NoAMF treatment group was significantly lower from the shoot length in the S2 AMF treatment group (Fig. 2A).

### Root length

The root length in the Control AMF treatment group was 26.5 ± 1.32, which was significantly higher than the root length in the S2 AMF treatment group (21 ± 1) by approximately 20.8%. The root length in the Control NoAMF treatment group was 24.33 ± 1.15, which was significantly higher from the root length in the S2 NoAMF treatment group (15 ± 4.44). These results suggest that AMF has a positive effect on root length in wheat, especially under normal soil conditions. Exposure to S2 had a negative effect on root length (Fig. 2B).

### Shoot fresh weight

The shoot fresh weight in the Control AMF treatment group was 7.04 ± 2.59, which was significantly higher than the shoot fresh weight in the Control NoAMF treatment group (3.42 ± 0.14) by approximately 105.3%. This indicates that the presence of AMF has a positive effect on shoot fresh weight. The shoot fresh weight in the S2 AMF treatment group was 2.79 ± 0.36, which was not significantly different from the shoot fresh weight in the Control NoAMF treatment group. However, S2 NoAMF treatment group (2.14 ± 0.31) did not show any significant change compared to S2 AMF treatment group for shoot fresh weight (Fig. 2C).

### Root fresh weight

The AMF treatment for S2 showed a significant decrease in root fresh weight, with a percentage decrease of approximately 79% compared to the control with AMF. Similarly, the S2 NoAMF treatment also resulted in a significant decrease in root fresh weight, compared to the control AMF. On the other hand, Control No AMF treatment resulted in a non-significant increase in root fresh weight than S2 NoAMF (Fig. 2D).

### Shoot dry weight

The AMF treatment for S2 showed a significant decrease in shoot dry weight, with a percentage decrease of approximately 54% compared to the control with AMF. Similarly, the S2 NoAMF treatment also resulted in a non-significant decrease in shoot dry weight, with a percentage decrease of about 28% compared to the control without AMF. On the other hand, the use of NoAMF treatment in S2 resulted in a non-significant decrease in shoot dry weight, compared to the S2 AMF (Fig. 2E).

### Root dry weight

The AMF treatment for S2 showed a significant decrease in root dry weight, with a percentage decrease of approximately 62% compared to the control with AMF. Similarly, the S2 NoAMF treatment also resulted in a significant decrease in root dry weight, with a percentage decrease of about 71% compared to the control without AMF. Compared to S2 NoAMF, AMF in the Control also resulted in a significant increase in root dry weight (Fig. 2F).

### Number of leaves per plant

**Figure 3:**
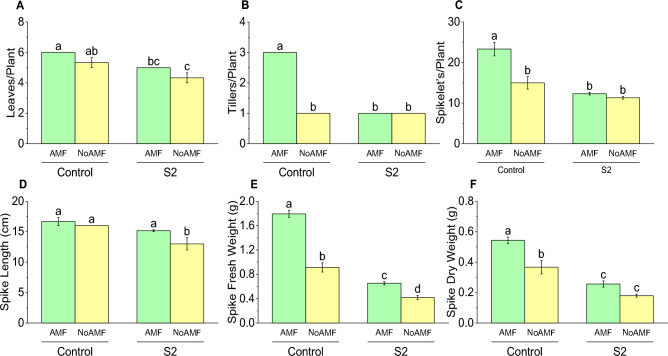
Effect of arbuscular mycorrhizal fungi (AMF) on leaves/plant (**A**), tillers/plant (**B**), spikelet’s/plant (**C**), spike length (**D**), spike fresh weight (**E**) and spike dry weight (**F**) in wheat cultivated under normal and saline soil conditions (200 mM NaCl). The data reported are the mean values ± standard error of three independent replicates. Statistical analysis using Fisher's least significant difference (LSD) test showed that significant differences (*p* < 0.05) existed among the treatment groups. The use of different letters to label the means indicates the presence of significant differences between the groups.

The control group plants inoculated with AMF had an average of 6.0 leaves per plant, which was a 12.5% increase compared to the control group plants without AMF inoculation, which had an average of 5.33 leaves per plant. Similarly, the S2 group plants inoculated with AMF had an average of 5.0 leaves per plant, which was a 16.67% increase compared to the S2 group plants without AMF inoculation, which had an average of 4.33 leaves per plant (Fig. 3A).

### Tillers per plant

The results indicate that there was a significant difference in the average number of tillers per plant between the AMF-inoculated and non-inoculated groups. The control group plants inoculated with AMF had an average of 3.0 tillers per plant, which was significantly higher than the average of 1.0 tiller per plant in the control group plants without AMF inoculation. Similarly, both S2 groups, AMF-inoculated and non-inoculated, had an average of 1.0 tiller per plant, with no significant difference between them (Fig. 3B).

### Spikelets per plant

The control group plants inoculated with AMF had an average of 23.33 spikelets per plant, which was significantly higher than the average of 15.0 spikelets per plant in the control group plants without AMF inoculation. Similarly, the S2 group plants inoculated with AMF had an average of 12.33 spikelets per plant, which was non-significantly higher than the average of 11.33 spikelets per plant in the S2 group plants without AMF inoculation. Furthermore, the results showed that the AMF-inoculated control group had a 55.56% increase in the average number of spikelets per plant compared to the control group without AMF inoculation. Similarly, the AMF-inoculated S2 group had a 8.83% increase in the average number of spikelets per plant compared to the non-inoculated S2 (Fig. 3C).

### Spike length

In Control AMF no significant change was noted compare to Control NoAMF for spike length. On the other hand, there was a significant increase in spike length in the control group without AMF treatment over S2 without AMF. In treatment group S2, the plants treated with AMF showed a 15.17% significant increase in spike length compared to the plants without AMF treatment (Fig. 3D).

### Spike fresh weight

The control group treated with AMF showed a significabt increase in spike fresh weight compared to the control group without AMF treatment. Conversely, the control group without AMF treatment showed a spike fresh weight of 0.91 g. In treatment group S2, the plants treated with AMF showed a spike fresh weight of 0.65 g, which was significantly higher (55%) than the spike fresh weight of the plants without AMF treatment (0.42 g) (Fig. 3E).

### Spike dry weight

The control group treated with AMF had a spike dry weight of 0.54 g, which was 46% higher than the control group without AMF treatment that had a spike dry weight of 0.37 g. Similarly, the S2 group treated with AMF had a spike dry weight of 0.26 g, which was 44% higher than the S2 group without AMF treatment that had a spike dry weight of 0.18 g (Fig. 3F).

### Chlorophyll a

**Figure 4:**
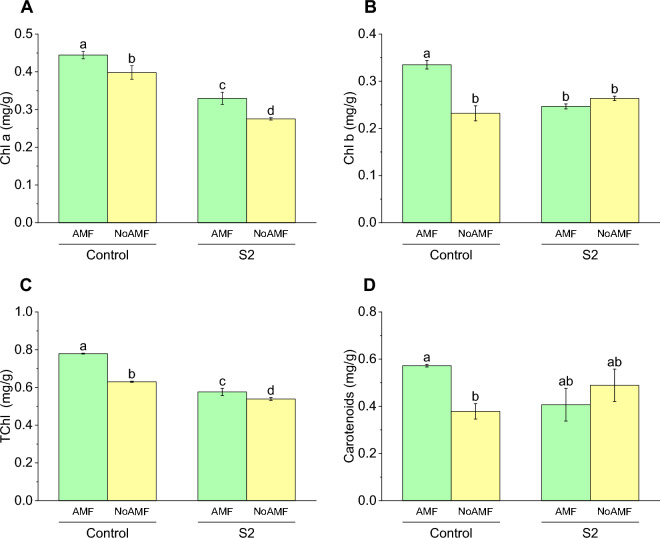
Effect of arbuscular mycorrhizal fungi (AMF) on contents of chlorophyll a (**A**), chlorophyll b (**B**), carotenoids (**C**), and total chlorophyll (**D**) in wheat cultivated under normal and saline soil conditions (200 mM NaCl). The data reported are the mean values ± standard error of three independent replicates. Statistical analysis using Fisher's least significant difference (LSD) test showed that significant differences (*p* < 0.05) existed among the treatment groups. The use of different letters to label the means indicates the presence of significant differences between the groups.

The mean concentration of chlorophyll a in the Control AMF treatment group was 0.44 ± 0.01686, which was approximately 13% higher than the mean concentration of chlorophyll a in the Control NoAMF treatment group (0.39 ± 0.03143). These findings suggest that the presence of AMF may promote chlorophyll a production in plants. The concentration of chlorophyll a in the S2 AMF treatment group was 0.32 ± 0.02804, which was lower than the concentration of chlorophyll a in the Control AMF treatment group. This indicates that the negative impact of S2 on chlorophyll a concentration is compounded by the presence of AMF. Similarly, the mean concentration of chlorophyll a in the S2 NoAMF treatment group was 0.27 ± 0.00577, which was significantly lower than the concentration of chlorophyll a in the Control NoAMF and S2 AMF treatment groups (Fig. 4A).

### Chlorophyll b

The concentration of chlorophyll b in the Control AMF treatment group was 0.33 ± 0.01572 (mean ± SD), which was approximately 43% higher than the mean concentration of chlorophyll b in the Control NoAMF treatment group (0.23 ± 0.02762). These findings suggest that the presence of AMF may promote chlorophyll b production in plants. Interestingly, the effect of S2 on chlorophyll b concentration was not consistent across the AMF treatments. The mean concentration of chlorophyll b in the S2 AMF treatment group was 0.24 ± 0.00924, which showed non-significantly change over S2 No AMF. However, the mean concentration of chlorophyll b in the S2 NoAMF treatment group (0.26 ± 0.00802) was slightly higher than the mean concentration of chlorophyll b in the Control NoAMF treatment group (0.23 ± 0.02762) by approximately 13% (Fig. 4B).

### Total chlorophyll

The concentration of total chlorophyll in the Control AMF treatment group was 0.77 ± 0.00404, which was significantly higher than the concentration of total chlorophyll in the Control NoAMF treatment group (0.63 ± 0.005) by approximately 22%. The concentration of total chlorophyll in the S2 AMF treatment group was significantly lower than the concentration of total chlorophyll in the Control AMF treatment group. The concentration of total chlorophyll in the S2 NoAMF treatment group was also significantly lower than the mean concentration of total chlorophyll in the Control NoAMF treatment group (Fig. 4C).

### Carotenoids

In the Control AMF treatment group, the concentration of carotenoids was 0.57 ± 0.0095, which was higher than the mean concentration in the Control NoAMF treatment group (0.38 ± 0.05686) by approximately 50%. The concentration of carotenoids in the S2 AMF treatment group was 0.41 ± 0.1202, which was not significantly different from the concentration in the Control AMF treatment group. The concentration of carotenoids in the S2 NoAMF treatment group was 0.49 ± 0.11877, which was not significantly higher from the S2 AMF and Control NoAMF treatment groups (Fig. 4D).

### APX activity

**Figure 5:**
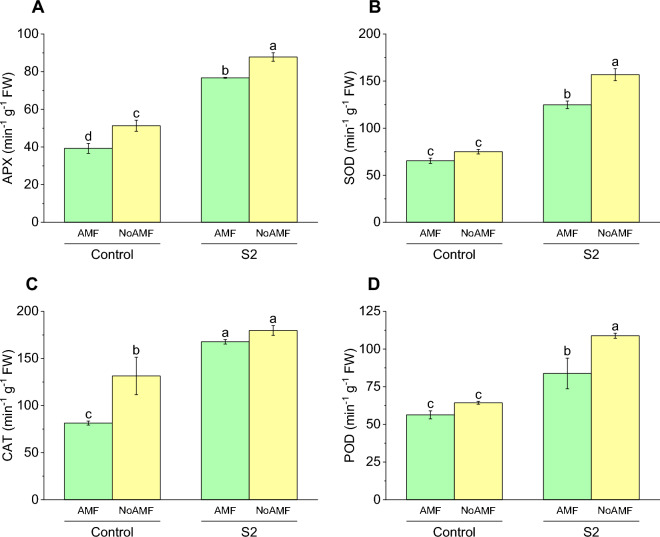
Effect of arbuscular mycorrhizal fungi (AMF) on the concentration of APX (**A**), SOD (**B**), CAT (**C**), and POD (**D**) in wheat cultivated under normal and saline soil conditions (200 mM NaCl). The data reported are the mean values ± standard error of three independent replicates. Statistical analysis using Fisher's least significant difference (LSD) test showed that significant differences (*p* < 0.05) existed among the treatment groups. The use of different letters to label the means indicates the presence of significant differences between the groups.

The highest APX activity was observed in the S2 NoAMF treatment group, with a mean value of 87.8 ± 3.89744, indicating a significant increase in APX activity compared to the Control AMF treatment group (39.26 ± 4.59384). The S2 AMF treatment group showed a mean APX activity of 76.73 ± 0.40415, which was approximately 95% higher than the Control AMF treatment group. These results suggest that exposure to S2 and the absence of AMF have a positive impact on APX activity in wheat. The Control NoAMF treatment group exhibited a mean APX activity of 51.3 ± 5.10979, which was approximately 30.6% higher than the Control AMF treatment group. These results suggest that the presence of AMF has a negative impact on APX activity in wheat under normal soil conditions (Fig. 5A).

### SOD activity

The mean activity of SOD in the Control AMF treatment group was 65.33 ± 4.85009, while in the Control NoAMF treatment group, it was 75.03 ± 4.17652. This resulting in approximately 15% lower SOD activity in the Control AMF treatment group compared to the Control NoAMF treatment group. The mean activity of SOD in the S2 AMF treatment group was 124.76 ± 6.99, which was significantly higher than that in the Control AMF treatment group by approximately 91%. The mean activity of SOD in the S2 NoAMF treatment group was 156.83 ± 11.00, which was significantly higher than that in the S2 AMF treatment group by approximately 25.7%. This suggests that the S2 alone, resulting in approximately 26% lower SOD activity in the S2 AMF treatment group compared to the S2 NoAMF treatment group (Fig. 5B).

### CAT activity

The Control AMF treatment group exhibited a mean CAT enzyme activity of 81.4 ± 3.65, which was significantly lower than the mean enzyme activity in the Control NoAMF treatment group (131.43 ± 34.33). On the other hand, the S2 AMF treatment group displayed a mean CAT enzyme activity of 167.73 ± 4.13, which was significantly higher than the mean enzyme activity in the Control AMF treatment group by approximately 106%. Furthermore, the mean CAT enzyme activity in the S2 NoAMF treatment group was 179.73 ± 9.07, which was significantly higher than the mean enzyme activity in the Control NoAMF treatment group by approximately 37%. This implies that S2 exposure alone has a greater positive impact on CAT enzyme activity than the absence of AMF alone (Fig. 5C).

### POD activity

The mean POD enzyme activity in the Control AMF treatment group was 56.33 ± 4.61, which was not significantly different from the mean enzyme activity in the Control NoAMF treatment group (64.33 ± 1.59). The mean POD enzyme activity in the S2 AMF treatment group was 83.8 ± 17.61, which was significantly higher than the mean enzyme activity in the Control AMF treatment group by approximately 49%. The mean POD enzyme activity in the S2 NoAMF treatment group was 108.83 ± 2.89, which was significantly higher than the mean enzyme activity in the Control NoAMF treatment group by approximately 69%. This suggests that S2 exposure alone has a greater positive effect on POD enzyme activity than the absence of AMF alone (Fig. 5D).

### H_2_O_2_ content

**Figure 6:**
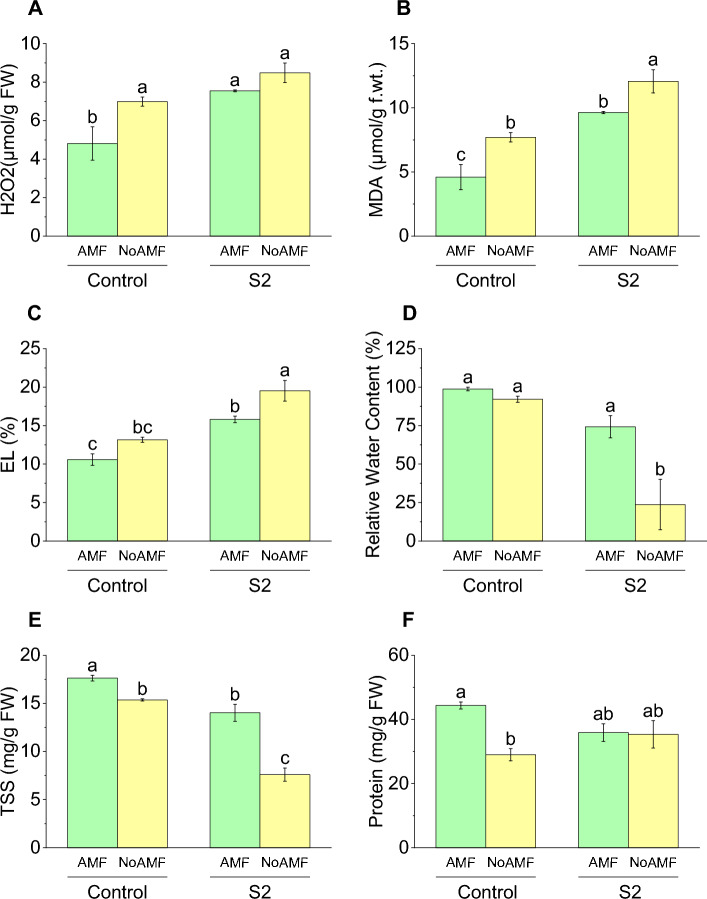
Effect of arbuscular mycorrhizal fungi (AMF) on H_2_O_2_ (**A**), MDA (**B**), electrolyte leakage (**C**), relative water contents (**D**), TSS (**E**) and protein (**F**) in wheat cultivated under normal and saline soil conditions (200 mM NaCl). The data reported are the mean values ± standard error of three independent replicates. Statistical analysis using Fisher's least significant difference (LSD) test showed that significant differences (*p* < 0.05) existed among the treatment groups. The use of different letters to label the means indicates the presence of significant differences between the groups.

The H_2_O_2_ content varied among the different treatments, with the highest value of 8.49 μmol/g observed in the S2 group without AMF treatment. This value was significantly higher than all other groups especially an increase compared to the control group treated with AMF that had a H_2_O_2_ content of 4.81 μmol/g. The control group without AMF treatment had a H_2_O_2_ content of 6.99 μmol/g, which was 45% higher than the control group treated with AMF. Similarly, the S2 group without AMF treatment had a H_2_O_2_ content of 8.49 μmol/g, which was 12% higher than the S2 group treated with AMF that had a H_2_O_2_ content of 7.55 μmol/g (Fig. 6A).

### MDA content

Results showed highest MDA content in the S2 group without AMF treatment, with a value of 12.07 µmol/g, which was higher than all other treatments. The Control group with AMF treatment had the second highest MDA content with a value of 7.7 µmol/g, which was higher than the control group treated with AMF, which had a MDA content of 4.6 µmol/g. This indicates that the application of AMF can potentially reduce the MDA content in plants, thereby reducing lipid peroxidation. The reduction in MDA content was 67% for the control group, where the MDA content was 4.6 µmol/g in AMF compared to 7.7 µmol/g without AMF treatment. Similarly, the S2 group treated with AMF had a lower MDA content of 9.63 µmol/g compared to the S2 group without AMF treatment that had a value of 12.07 µmol/g. The reduction in MDA content was 25% for the S2 group, where the MDA content was 9.63 µmol/g in the group treated with AMF compared to 12.07 µmol/g in the S2 group without AMF treatment (Fig. 6B).

### Electrolyte leakage

The highest electrolyte leakage was observed in the S2 group without AMF treatment, with a value of 19.52%, which was significantly higher than all other groups. This suggests that the absence of AMF treatment may have led to increased membrane damage, resulting in higher electrolyte leakage in the plants. The control group without AMF treatment had the second highest electrolyte leakage with a value of 13.16%, which was significantly higher than the control group treated with AMF, which had an electrolyte leakage of 10.58%. This indicates that the application of AMF can potentially reduce membrane damage, thereby reducing electrolyte leakage in plants. The reduction in electrolyte leakage was 24% for the control group, where the electrolyte leakage was 10.58% in the group treated with AMF compared to 13.16% in the control group without AMF treatment. Similarly, the S2 group treated with AMF had a lower electrolyte leakage of 15.81% compared to the S2 group without AMF treatment that had a value of 19.53%. The reduction in electrolyte leakage was 23% for the S2 group, where the electrolyte leakage was 15.81% in the group treated with AMF compared to 19.53% in the S2 group without AMF treatment (Fig. 6C).

### Relative water content

The highest RWC was observed in the control group treated with AMF, with a value of 98.79%, which was significantly higher than all other groups. This suggests that the application of AMF can potentially improve the water status of plants, resulting in higher RWC. The control group without AMF treatment had a lower RWC of 92.09%, which was non-significantly lower than the control group treated with AMF. This indicates that the absence of AMF treatment may have led to a reduction in water status, resulting in lower RWC in the plants. The increase in RWC was 7% for the control group, where the RWC was 98.79% in the group treated with AMF compared to 92.09% in the control group without AMF treatment. Similarly, the S2 group treated with AMF had a higher RWC of 74.24% compared to the S2 group without AMF treatment that had a value of 23.70%. The increase in RWC was 213% for the S2 group, where the RWC was 74.24% in the group treated with AMF compared to 23.70% in the S2 group without AMF treatment (Fig. 6D).

### Total soluble solids (TSS)

The control group treated with AMF had the highest Total soluble solids (TSS) value of 17.63 mg/g FW, which was significantly higher than all other groups. The control group without AMF treatment had a TSS value of 15.37 mg/g FW, which was lower than the control with AMF. The increase in TSS was 14.7% for the control group, with AMF compared to control NoAMF treatment. Similarly, the S2 group treated with AMF had a TSS value of 14.03 mg/g FW, which was higher than the S2 group without AMF treatment that had a TSS value of 7.6 mg/g FW. The increase in TSS was 85% for the S2 AMF compared to S2 No AMF treatment (Fig. 6E).

### Protein contents

The highest protein content was found in the Control AMF group with a mean value of 44.37 mg/g FW, which was significantly higher than the other groups. The Control NoAMF group had a mean protein content of 29.00 mg/g FW, which was significantly lower than the Control AMF group. The S2 AMF group had a mean protein content of 35.90 mg/g FW, which was slightly lower than the Control AMF group but non-significantly higher than the Control NoAMF group. Finally, the S2 NoAMF group had a mean protein content of 35.37 mg/g FW, which was similar to the S2 AMF group. The Control NoAMF group showed a 34.6% decrease in protein content compared to the Control AMF group (Fig. 6F).

**Figure 7:**
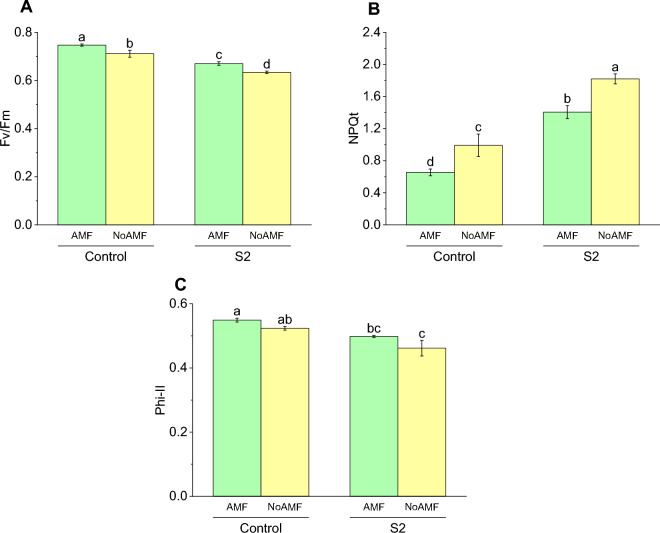
Effect of arbuscular mycorrhizal fungi (AMF) on Fv/Fm (**A**), NPQt (**B**) and Phi-II (**C**) in wheat cultivated under normal and saline soil conditions (200 mM NaCl). The data reported are the mean values ± standard error of three independent replicates. Statistical analysis using Fisher's least significant difference (LSD) test showed that significant differences (*p* < 0.05) existed among the treatment groups. The use of different letters to label the means indicates the presence of significant differences between the groups.

For the control group, the mean Fv/Fm with AMF was 0.75 cm (± 0.008 SD), which was a 5% increase compared to the mean Fv/Fm without AMF (0.711 cm ± 0.02506 SD). For the experimental group (S2), the mean Fv/Fm with AMF was 0.67 cm (± 0.013 SD), which was a 6% increase compared to the mean Fv/Fm without AMF (0.63 cm ± 0.00929 SD) (Fig. 7A).

The results showed that the NPQt values varied significantly among the treatments. For the control group, plants without AMF had a mean NPQt of 0.99 (± 0.24233 SD), which was significantly higher than plants with AMF, which had a mean NPQt of 0.65 (± 0.07253 SD). For the experimental group (S2), plants without AMF had a mean NPQt of 1.82 (± 0.1079 SD), which was significantly higher than plants with AMF, which had a mean NPQt of 1.40 (± 0.13944 SD) (Fig. 7B).

Specifically, for the control group, plants treated with AMF had a mean Phi-II value of 0.54 (± 0.01015 SD), which was slightly higher than the mean Phi-II value of 0.52 (± 0.01012 SD) for plants without AMF. Similarly, for the experimental group (S2), plants treated with AMF had a mean Phi-II value of 0.50 (± 0.00551 SD), which was also slightly higher than the mean Phi-II value of 0.46 (± 0.04196 SD) for plants without AMF (Fig. 7C).

### Shoot Zn concentration

**Figure 8:**
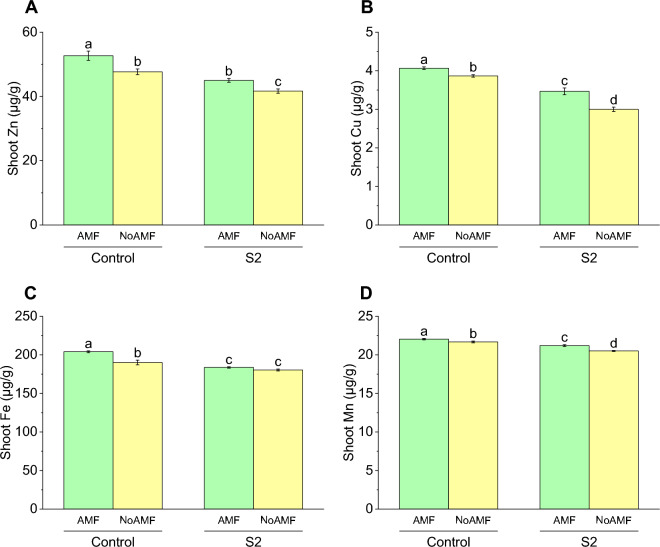
Effect of arbuscular mycorrhizal fungi (AMF) on shoot Zn (**A**), shoot Cu (**B**) shoot Fe (**C**) and shoot Mn (**D**) in wheat cultivated under normal and saline soil conditions (200 mM NaCl). The data reported are the mean values ± standard error of three independent replicates. Statistical analysis using Fisher's least significant difference (LSD) test showed that significant differences (*p* < 0.05) existed among the treatment groups. The use of different letters to label the means indicates the presence of significant differences between the groups.

The highest shoot Zn concentration was observed in the control AMF treated plants with a mean value of 52.67 µg/g, while the lowest concentration was found in the S2 NoAMF plants with a mean value of 41.67 µg/g. The shoot Zn concentration in the control NoAMF and S2 AMF plants was 47.67 µg/g and 45 µg/g, respectively. Compared to the control NoAMF plants, the shoot Zn concentration in the control AMF plants increased by approximately 11%. Similarly, the shoot Zn concentration in the S2 AMF plants increased by 8% compared to the S2 NoAMF plants. However, the shoot Zn concentration in the S2 NoAMF plants decreased by about 12.5% compared to the control NoAMF plants (Fig. 8A).

### Shoot Cu concentration

The shoot Cu concentration (µg/g) was determined for the control and treatment groups. The results showed that the mean shoot Cu concentration in the AMF-treated control group was 4.07 µg/g, which was slightly higher than that of the NoAMF-treated control group (3.87 µg/g). This indicates a 5.2% increase in Cu concentration in the AMF-treated group compared to the control group without AMF treatment. In the S2 treatment groups, the shoot Cu concentration was lower than that of the control groups. The mean shoot Cu concentration in the AMF-treated S2 group was 3.47 µg/g, which represents a 17.2% decrease compared to the AMF-treated control group. The NoAMF-treated S2 group had the lowest mean shoot Cu concentration of 3.0 µg/g, indicating a 29% decrease compared to the NoAMF-treated control group (Fig. 8B).

### Shoot Fe concentration

The results showed that the highest shoot Fe concentration was found in the control with AMF treatment, with an average value of 204 µg/g. This was followed by the control without AMF treatment, with an average value of 190 µg/g. On the other hand, the S2 with AMF and S2 without AMF treatments showed lower shoot Fe concentrations, with average values of 183.67 µg/g and 180.33 µg/g, respectively. Compared to the control with AMF treatment, the control without AMF treatment showed a decrease in shoot Fe concentration by 7.36%, while the S2 with AMF and S2 without AMF treatments showed a decrease of 11% and 13%, respectively (Fig. 8C).

### Shoot Mn concentration

In terms of shoot Mn concentration (µg/g), the highest value was observed in the Control AMF treatment with 22.03 µg/g, followed closely by the Control NoAMF treatment with 21.67 µg/g. The S2 AMF treatment had a shoot Mn concentration of 21.2 µg/g, while the S2 NoAMF treatment had the lowest value with 20.5 µg/g. Compared to the Control AMF treatment, the Control NoAMF treatment showed a slight decrease in shoot Mn concentration by 1.7%, while S2 AMF treatment showing a decrease of 3.9% and the S2 NoAMF treatment showing a decrease of 7.4% (Fig. 8D).

### Root Zn concentration

**Figure 9:**
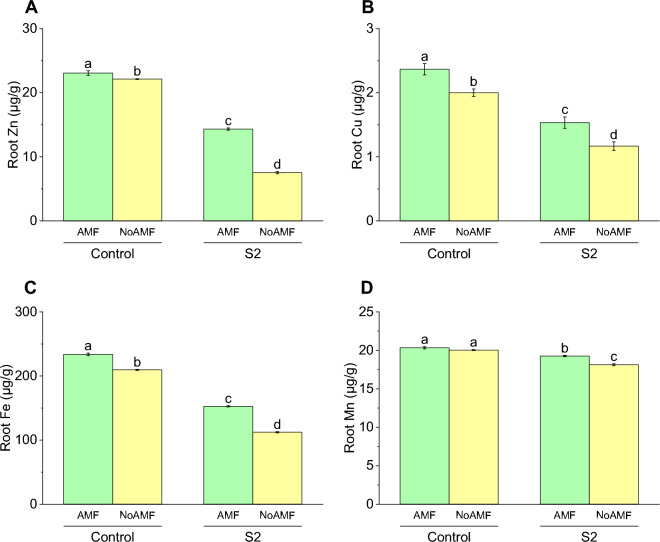
Effect of arbuscular mycorrhizal fungi (AMF) on root Zn (**A**), root Cu (**B**) root Fe (**C**) and root Mn (**D**) in wheat cultivated under normal and saline soil conditions (200 mM NaCl). The data reported are the mean values ± standard error of three independent replicates. Statistical analysis using Fisher's least significant difference (LSD) test showed that significant differences (*p* < 0.05) existed among the treatment groups. The use of different letters to label the means indicates the presence of significant differences between the groups.

The root Zn concentration was significantly higher in the AMF-treated plants compared to the NoAMF plants in both the Control and S2 groups. Specifically, the Control AMF group had a mean root Zn concentration of 23.07 µg/g, which was 4% higher than the Control NoAMF group with a mean concentration of 22.13 µg/g. Similarly, the S2 AMF group had a mean root Zn concentration of 14.33 µg/g, which was 90% higher than the S2 NoAMF group with a mean concentration of 7.53 µg/g. These results suggest that AMF treatment can increase root Zn concentration, particularly in plants grown under stressful conditions (Fig. 9A).

### Root Cu concentration

The root Cu concentration in the Control group with AMF treatment was 2.37 µg/g, while without AMF treatment it was 2.0 µg/g with a standard deviation of 0.1 µg/g. This represents a 18.5% increase in Cu concentration with AMF treatment compared to without AMF. For the S2 group, the root Cu concentration with AMF treatment was 1.53 µg/g, while without AMF treatment it was 1.17 µg/g. This represents a 31% increase in Cu concentration with AMF treatment compared to without (Fig. 9B).

### Root Fe concentration

The results showed that the Control AMF group had the highest root Fe concentration at 233.67 µg/g. The Control NoAMF group had a decrease of 11.48% in root Fe concentration compared to the Control AMF group, with a value of 209.67 µg/g. The S2 AMF and S2 NoAMF groups had even greater decreases in root Fe concentration, with values of 152.67 µg/g and 112.33 µg/g, respectively (Fig. 9C).

### Root Mn concentration

The Control AMF group had a concentration of 20.33 µg/g, while the Control NoAMF group had a slightly lower concentration of 20.03 µg/g. The S2 AMF group had a concentration of 19.27 µg/g, which was a 5.5% decrease compared to the Control AMF group. The S2 NoAMF group had the lowest Mn concentration of 18.13 µg/g, which was a 12% decrease compared to the Control AMF group (Fig. 9D).

### Shoot Na concentration

**Figure 10:**
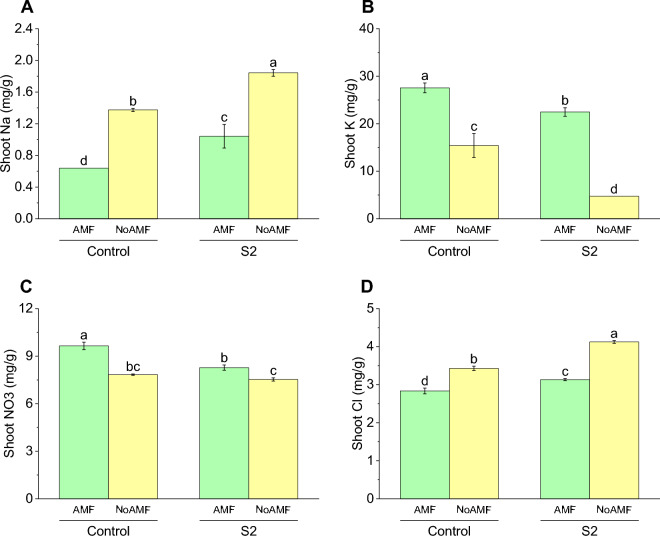
Effect of arbuscular mycorrhizal fungi (AMF) on shoot Na (**A**), shoot K (**B**) shoot NO_3_ (**C**) and shoot Cl (**D**) in wheat cultivated under normal and saline soil conditions (200 mM NaCl). The data reported are the mean values ± standard error of three independent replicates. Statistical analysis using Fisher's least significant difference (LSD) test showed that significant differences (*p* < 0.05) existed among the treatment groups. The use of different letters to label the means indicates the presence of significant differences between the groups.

The results show that the Control AMF group had the lowest shoot Na concentration at 0.64 mg/g, while the Control NoAMF group had a significant increase with a concentration of 1.37 mg/g. The S2 AMF group had a concentration of 1.04 mg/g, which increased compared to S2 NoAMF group with a concentration of 1.84 mg/g (Fig. 10A).

### Shoot K concentration

The Control AMF group had a mean concentration of 27.54 mg/g, the Control NoAMF group had a mean concentration of 15.42 mg/g, the S2 AMF group had a mean concentration of 22.47 mg/g, and the S2 NoAMF group had a mean concentration of 4.75 mg/g. Interestingly, the Control NoAMF group had a significant decrease in shoot K concentration compared to the Control AMF group, indicating a potential negative effect of the absence of AMF. In contrast, the S2 AMF group had a slightly lower shoot K concentration compared to the Control AMF group, while the S2 NoAMF group had a much greater decrease (Fig. 10B).

### Shoot NO_3_ concentration

The shoot NO_3_ content of tomato plants was significantly affected by both the AMF inoculation and the S2 treatment. The Control AMF group had the highest shoot NO_3_ content, with a mean value of 9.65 mg/g. The Control NoAMF group had a lower shoot NO_3_ content of 7.83 mg/g, representing a decrease compared to the Control AMF group. The S2 AMF group had a mean shoot NO_3_ content of 8.28 mg/g, while the S2 NoAMF group had the lowest shoot NO_3_ content at 7.53 mg/g, representing a 9.96% decrease compared to the S2 AMF group. These results suggest that both AMF inoculation and the S2 treatment can have significant effects on the shoot NO_3_ content of tomato plants (Fig. 10C).

### Shoot Cl concentration

The shoot Cl concentration was measured for all treatment groups, with the Control AMF group having a concentration of 2.84 mg/g. The Control NoAMF group had a slightly higher concentration of 3.43 mg/g, indicating a 20.77% decrease in Cl concentration compared to the Control AMF group. The S2 AMF group had a concentration of 3.13 mg/g. The S2 NoAMF group had the highest concentration of shoot Cl at 4.13 mg/g, showing a 45% increase compared to the Control AMF group. These results suggest that the absence of AMF in the soil may lead to an increase in Cl accumulation in the shoot tissues of plants (Fig. 10D).

### Root Na concentration

**Figure 11:**
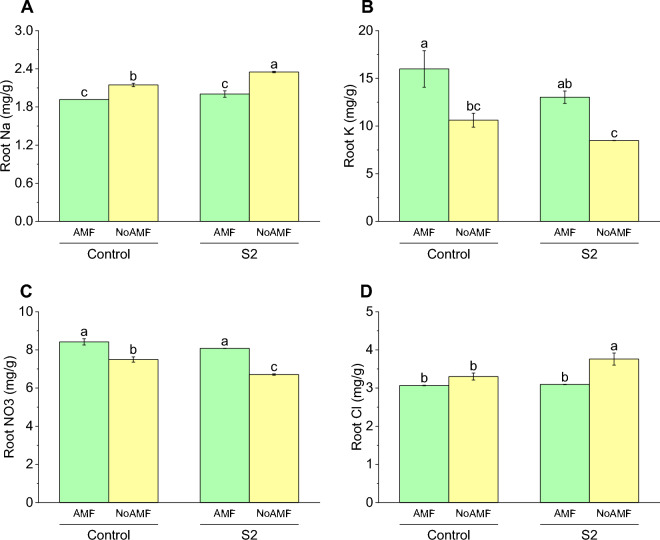
Effect of arbuscular mycorrhizal fungi (AMF) on root Na (**A**), root K (**B**) root NO_3_ (**C**) and root Cl (**D**) in wheat cultivated under normal and saline soil conditions (200 mM NaCl). The data reported are the mean values ± standard error of three independent replicates. Statistical analysis using Fisher's least significant difference (LSD) test showed that significant differences (*p* < 0.05) existed among the treatment groups. The use of different letters to label the means indicates the presence of significant differences between the groups.

The Control AMF group had the lowest Na concentration at 1.92 mg/g, while the Control NoAMF group had a slightly higher Na concentration of 2.15 mg/g. The S2 AMF group had a Na concentration of 2.00 mg/g, which was not significantly different from the Control AMF group. Finally, the S2 NoAMF group had the highest Na concentration at 2.35 mg/g (Fig. 11A).

### Root K concentration

The results for root K concentration showed that all treatments resulted in a significant increase in K concentration compared to the Control NoAMF group. The Control AMF group had the highest concentration at 15.99 mg/g with an increase compared to the Control NoAMF group at 10.61 mg/g. The S2 AMF group had a concentration of 13.02 mg/g, which showed an increase compared to the Control NoAMF group, while the S2 NoAMF group had the lowest concentration at 8.49 mg/g, still showing an increase compared to the Control NoAMF group (Fig. 11B).

### Root NO_3_ concentration

For root NO_3_ concentration, it was found that the Control AMF group had an average concentration of 8.42 mg/g, while the Control NoAMF group had an average concentration of 7.50 mg/g. The S2 AMF group showed a slight increase in root NO_3_ concentration compared to the Control AMF group, with an average concentration of 8.08 mg/g. On the other hand, the S2 NoAMF group had a lower root NO_3_ concentration compared to the Control AMF and NoAMF groups, with an average concentration of 6.71 mg/g (Fig. 11C).

### Root Cl concentration

The root Cl concentration results showed that the highest mean value was observed in the S2 NoAMF treatment (3.76 mg/g), which was significantly higher than all other treatments. The lowest mean value was found in the Control AMF treatment (3.08 mg/g), which was significantly lower than the S2 NoAMF treatment but not significantly different from the other two treatments. The mean values for the Control NoAMF and S2 AMF treatments were 3.30 mg/g and 3.09 mg/g, respectively. Compared to the control AMF treatment, the Control NoAMF and S2 AMF treatments showed a slight increase in root Cl concentration, but the difference was not statistically significant (Fig. 11D).

### Principal component analysis

The loadings table shows the loadings or coefficients of each variable on the principal components (PCs) generated by the analysis. In this case, the PCA generated two PCs, PC1 and PC2, which together account for 84.2% of the total variation in the data (PC1 accounts for 75.2% and PC2 accounts for 9.0%). The loadings table presents the loadings of each variable on both PCs. The first column of the table lists the variables analyzed, including plant growth parameters, chlorophyll fluorescence parameters, mineral nutrient concentrations, and antioxidant enzyme activities. The next four columns present the loadings of each variable on PC1 and PC2. The values in these columns represent the correlation coefficients between the variables and the PCs, with positive values indicating a positive correlation and negative values indicating a negative correlation. Looking at the loadings, we can see that the variables with the highest loadings on PC1 include shoot dry weight, shoot length, root dry weight, root fresh weight, shoot fresh weight, and total chlorophyll. These variables are positively correlated with each other and with PC1, which likely represents overall plant growth and development. On the other hand, variables with high loadings on PC2 include NPQt, and Chl b. These variables are positively correlated with each other and with PC2, which may represent variations in chlorophyll fluorescence parameters. In summary, the PCA results suggest that the variables analyzed can be grouped into two main categories: those related to overall plant growth and development and those related to chlorophyll fluorescence parameters. These results can be useful for identifying the most important variables affecting plant growth and development and for exploring the underlying physiological mechanisms (Table 2; Figures S1–S2).

**Table 2:** Principal componentant analysis loadings values for growth, chlorophyll contents, antioxidents, nutrients concentration in root and shoot and biochemical attributes.

Loadings	PC1 (75.2%)	PC2 (9.0%)
	Salinity and AMF	
	Loadings	Loadings
Leaves/plant	0.15324	− 0.01703
Shoot length (cm)	0.15173	0.08763
Root length (cm)	0.13364	− 0.07244
Tillers/plant	0.13573	0.24762
Spike length (cm)	0.14227	− 0.1227
Spikelet’s/plant	0.15133	0.11624
Shoot fresh weight (g)	0.12716	0.21721
Root fresh weight (g)	0.1353	0.17181
Spike fresh weight (g)	0.15974	0.10763
Shoot dry weight (g)	0.12082	0.22384
Root dry weight (g)	0.14495	0.13983
Spike dry weight (g)	0.1614	0.03648
Phi-II	− 0.16192	0.05001
NPQt	0.09463	0.35658
Fv/Fm	0.04939	0.38758
Chl a (mg/g)	0.07294	0.30146
Chl b (mg/g)	− 0.15535	0.08596
TChl (mg/g)	− 0.15253	0.13362
Carotenoids (mg/g)	− 0.15454	− 0.07154
TSS	− 0.15464	− 0.01192
Protein	− 0.15111	− 0.03426
SOD	− 0.16154	0.01562
POD	− 0.16009	0.06355
CAT	0.14568	− 0.17199
APX	0.15643	− 0.14637
H_2_O_2_(µmol/g FW)	0.15998	− 0.04477
Shoot Zn	0.16202	− 0.01486
Shoot Cu	0.15961	− 0.10045
Shoot Fe	0.15864	0.08947
Shoot Mn	0.16147	− 0.09898
Root Zn	0.1525	− 0.11244
Root Cu	0.16232	− 0.04018
Root Fe	0.15826	− 0.04883
Root Mn (µg/g)	0.15717	− 0.13337
Shoot Na	− 0.14706	0.02989
Shoot K	0.1427	− 0.09365
Shoot NO_3_ (mg/g)	0.14585	0.09941
Shoot Cl	− 0.14751	0.10708
Root Na	− 0.14082	0.09593
Root K	0.1339	− 0.05424
Root NO_3_	0.14013	− 0.09748
Root Cl	− 0.12814	0.1589

Principal Component Analysis (PCA) was conducted on the given dataset containing observations, PC1 scores, PC2 scores, and group scores. PC1 and PC2 explain 75.2% and 9.0% of the total variance in the dataset, respectively. The observations were divided into three groups, namely AMF, NoAMF, and Control, based on their group scores. Observations in the AMF group have positive PC1 scores, ranging from 8.88 to − 2.53. This indicates that these observations have higher values in variables that contribute to PC1, such as plant growth or biomass. The AMF group observations also have PC2 scores ranging from 0.31 to − 2.85, indicating a range of values in variables contributing to PC2, such as root length or branching. The NoAMF group observations, on the other hand, have negative PC1 scores ranging from − 9.17 to 2.46, indicating lower values in variables contributing to PC1. The NoAMF group observations also have negative PC2 scores ranging from − 2.44 to − 0.88, indicating lower values in variables contributing to PC2. Finally, the Control group observations have positive PC1 scores ranging from 8.88 to 0.16, similar to the AMF group, indicating higher values in variables contributing to PC1. The Control group observations also have negative PC2 scores ranging from − 2.28 to − 0.88, similar to the NoAMF group, indicating lower values in variables contributing to PC2 (Table 3).

**Table 3:** Principal componentant analysis score values for AMF and salinity.

Observations	PC1 (75.2%)	PC2 (9.0%)	Group
Scores	Scores	Scores	Scores
AMF	8.87843	0.31175	1
AMF	8.13465	1.97824	1
AMF	7.36099	3.02866	1
AMF	− 0.6378	− 2.13844	1
AMF	− 1.91421	− 2.85289	1
AMF	− 2.53309	0.0434	1
NoAMF	2.4638	− 2.28407	2
NoAMF	1.03312	− 2.43865	2
NoAMF	0.16307	− 0.88273	2
NoAMF	− 6.11643	1.61974	2
NoAMF	− 7.659	2.30493	2
NoAMF	− 9.17352	1.31005	2
Control	8.87843	0.31175	1
Control	8.13465	1.97824	1
Control	7.36099	3.02866	1
S2	− 0.6378	− 2.13844	2
S2	− 1.91421	− 2.85289	2
S2	− 2.53309	0.0434	2
Control	2.4638	− 2.28407	1
Control	1.03312	− 2.43865	1
Control	0.16307	− 0.88273	1
S2	− 6.11643	1.61974	2
S2	− 7.659	2.30493	2
S2	− 9.17352	1.31005	2

## Discussion

Salinity is a significant environmental challenge that negatively impacts crop productivity. Wheat is a major cereal crop worldwide, and finding ways to enhance its productivity in salty conditions through mycorrhizae is crucial for ensuring food security. In the current study, salinity significantly negatively impacted wheat plants' output. The soil's salinity negatively impacts plant number and yield of grains. These findings agree with^52,53^. In our study, salt stress dramatically decreased the root, leaf dry matter, stem, and leaf area compared to the standard treatments. This was likely due to the direct impacts of ion harmfulness or the indirect effects of salt ions that create an osmotic imbalance between the soil and plants. These outcomes support the conclusions of^54,55^. Colonization with arbuscular mycorrhizal fungi (AMF) increased dry matter and leaf area of the salt-stressed plants. This effect of AMF on the dry matter was further prominent in aerial biomass compared to root biomass due to the higher allocation of carbohydrates to shoot tissues than root tissues^56^. The enhancement of host plant phosphorus (P) nutrition by mycorrhiza has been partly attributed to the improved development of mycorrhizal wheat plants development in saline conditions^57^. In the past^58^, discovered that promoting the host plant's root growth is one of Glomus sp.-induced salt stress amelioration methods. Although it's possible that this was due to the short growth period following transplantation, possible impacts of AMF on root biomasses under mild salt stress and root: shoot (R:S) ratios were not evident. Overall, neither salt stress nor transplanting AMF+ or AMF' seedlings showed any discernible differences in root-to-shoot ratios. However^59^, discovered that the AM fungi's effect on tomato dry matter accumulation was more prominent on above-ground biomass than on root biomass, altering the R:S ratio. Because several enzymes necessary for the production of photosynthetic pigments were suppressed in the current investigation, chlorophyll concentrations were considerably decreased by salt treatments, supporting the findings of^53,60^. Wheat plants have been found to have more chlorophyll in their leaves while growing in saline environments, supporting the findings of^26^. The antagonistic result of Na on Mg absorption is balanced and decreased in the occurrence of mycorrhiza^61^. The ability of mycorrhization to reverse stress in this method is shown by the fact that inoculation plants below salt stress attain amounts of photosynthetic capability that are even higher than those of non-stressed plants^53,62^. Chlorophyll (Chl) component content and Chla/Chlb ratio are crucial markers for determining the physiological state of plant photosynthetic tissues because they greatly impact plant photosynthesis^63^. Our findings support the conclusions^64^ that AMF had a favorable influence on the photosynthetic pigments (Chla and Chlb) content. Mesophyll cells contain photosynthetic pigments, which makes them more susceptible to salinity stress than most highly secured oxidases^65^. According to the current study, salt stress suppresses the action of chlorophyll synthase while increasing the enzyme's activity that degrades chlorophyll, causing plants under salt stress to have less chlorophyll^66^. Under salt stress, AMF inoculation can retain the K^+^/Na^+^ stability and boost plant photosynthetic capability, confirming the findings^65^. In the current work, chlorophyll fluorescence indicates early photochemical reactions in PSII and variations in the texture in addition state of photosynthetic locations, demonstrating plant adaptation to various environments and providing strategies for choosing salt-tolerant plants species. The electron transport chain in fenugreek chloroplasts was disrupted by salt stress, and increased ROS formation resulted in oxidative cell membrane system damage, according to^67^ research^68^. In contrast to the NM treatment, the M treatment boosted activity in Fv/Fm, and Fv/Fo decreased damage to the photosynthetic systems of E. Angustifolia leaves^65^. In our investigation, salt-affected plants had significantly lower phosphorus concentrations than control plants. Due to the rainfall of H_2_PO_4_ with Ca^2+^ ions in the earth, the competition of K and Ca using Na, P absorption in saline soils decreased^59^. AMF significantly affected P absorption even in the control plants. One of the main factors contributing to the enhanced development of salt-affected plants colonized by AMF is that AMF has been found to enhance plant P uptake^69,70^. In the current study, plants exposed to higher salinity accumulated less K^69^. At both salinity levels, mycorrhizal G. mosseae plants showed greater K concentrations. Researchers looked at various salt stress and also determined that Acacia nilotica AM fungal-inoculant had advanced K contents in shoots plus roots at all salt stress. Via sustaining a great K/Na ratio, changing plant cytoplasmic ionic balance, or increasing Na outflow from plants, advanced K deposition by mycorrhizal plants in salty soil may be advantageous^59,69^. Regardless of salt level, our study found that mycorrhizal plants had lower Na concentrations than nonmycorrhizal plants. The observation confirms the results that the loss of sensitivity of Na concentration to AMF treatments could be due to the weakened impacts of plant development stimulation produced by AMF colonization^59^. The antioxidant enzymes CAT, SOD, APX, and POD were shown to be more active in tomato plants in the current study when exposed to salt (excluding CAT also SOD activity at 100 mM NaCl). Conversely, as indicated by the concurrent rise of MDA, this increased activity did not offer sufficient defense against ROS. In the tomato plants used in this study, AMF considerably improved the action of antioxidant defense enzymes. In plant-pathogen interactions, superoxide radicals are created as the hypersensitive response develops; these findings are comparable to^26,59^. The antioxidant enzymes SOD, CAT, POD, and APX were found to be more active in tomato plants in the current study when exposed to salt (excluding CAT also SOD activity at 100 mM NaCl). These elevated activities could not offer sufficient defenses against ROS, as seen in the contemporaneous MDA increase. AMF considerably boosted the activity of the antioxidant defense enzymes in the tomato plants employed in this investigation. In plant-pathogen interactions, superoxide radicals are created as the hypersensitive response develops; these findings are comparable to^26,59^. The deficient dry weight (plant growth) is responsible for the sharp decline in the absorption of micronutrients by growing plants in highly salinized soil. Although the AMF inoculation somewhat boosted the uptake of micronutrients beneath the tremendously salty soil, the increase fell short of the values attained below the faintly as well as reasonably salt in the soil. This supports the conclusions of^71^ and suggests that the AMF inoculation under the highly salinized soil did not entirely accommodate for the adverse effects of the salinity on plant growth as well as nutrient absorption, but rather that it can somewhat improve plant development under such stressful conditions. AM injection led to an increase in Mn and Cu uptake. However, only at the 10% chance level was this increase substantial. Only plants cultivated in soil with the greatest salinity level benefited from adding P to improve Cu uptake. On the other hand, AM inoculation increased Zn uptake at all soil salinity levels. The current study suggests that increased nutrient uptake by AMF in saline-tension wheat plants may mitigate the detrimental impact of Cl-ions also Na^+^ by preserving the ions that cannot interfere with metabolism because of the vacuolar membrane's ability to compartmentalize and allow for targeted ion absorption. Tension from salt causes plant tissues to accumulate more Na^+^. This problem can be somewhat resolved using AM, reducing the number of Na^+^ ions present. The diluting effect brought on by growth enhancement could account for the reduction in Na^+^ content in mycorrhizal plants associated with non-AMF plants, which supports the findings of^72^. The general mechanism of AMF's relief of salt stress in wheat may include the inhibition of Na^+^ accretion and an increase in K^+^ attention^27^. In this study, the decreased buildup of lipid peroxidation, suggesting lesser oxidative stress in the occupied plants, was related to increased antioxidant enzyme activity in mycorrhizal plants as opposed to nonmycorrhizal plants. Based on the findings provided here, our findings are consistent with the idea that AMF can help defend plants alongside salinity by reducing the oxidative stress brought on by salt. This mycorrhizal colonization's ameliorative function reveals important relationships with cultivar and salt exposure. In mycorrhizal plants, increased antioxidant enzyme activity and decreased lipid peroxidation may help maintain the ion stability necessary for photochemical reactions in leaves underneath salt.

## Conclusion

The information above suggests that mycorrhizal symbiosis can provide ecosystem services to ensure plant production in saline soils. By increasing comparative water content also the membrane constancy index, improving photosynthetic proficiency as well as protein synthesis, causing a better osmotic adaptation through the accretion of compatible solutes, improving plant nutrient uptake, and preventing oxidative stress by lowering membrane lipid peroxidation and H_2_O_2_ content, it lessened the harmful effects of salt stress on plant efficiency. As a result, beneficial processes are functioning, which proposes that fostering this symbiotic relationship might benefit wheat plants in adjusting to salt stress. Based on our research, we can say that mycorrhizal colonization can promote carbon plus nitrogen absorptions in salt stress, resulting in higher grain yields and higher grain quality. These findings might have important practical ramifications since they show the potential of using AMF treatment in maintainable cultivation in arid and semi-arid environments. The current study adds fresh knowledge on AMF's plant growth promotion in saline soils.

## Supplementary Information

Supplementary Figures.

## Data Availability

The data generated or analyzed during this study are with this article.

## References

[1] Yousaf M.J. (2021). Cadmium source identification in soils and high-risk regions predicted by geographical detector method. Environ. Pollut..

[2] Soothar MK (2021). Assessment of acidic biochar on the growth, physiology and nutrients uptake of maize (*Zea mays* L.) seedlings under salinity stress. Sustainability.

[3] Arora NK (2019). Impact of climate change on agriculture production and its sustainable solutions. Environ. Sustain..

[4] Acquaah G (2007). Principles of Plant Genetics and Breeding.

[5] Sabagh, A. E. L. *et al.* Consequences and mitigation strategies of heat stress for sustainability of soybean (*Glycine max* L. Merr.) production under the changing climate. In *Plant Stress Physiology [Working Title]* (IntechOpen, 2020). 10.5772/intechopen.92098.

[6] Rivelli AR, James RA, Munns R, Condon AG (2002). Effect of salinity on water relations and growth of wheat genotypes with contrasting sodium uptake. Funct. Plant Biol..

[7] Farooq MU (2020). Effect of silicon and gibberellic acid on growth and flowering of gladiolus. World J. Biol. Biotechnol..

[8] Bose S (2018). Effects of salinity on seedling growth of four maize (*Zea mays* L.) cultivars under hydroponics. J. Agric. Stud..

[9] Saboor A (2021). Biofertilizer-based zinc application enhances maize growth, gas exchange attributes, and yield in zinc-deficient soil. Agriculture.

[10] Alam MM, Nahar K, Hasanuzzaman M, Fujita M (2014). Exogenous jasmonic acid modulates the physiology, antioxidant defense and glyoxalase systems in imparting drought stress tolerance in different Brassica species. Plant Biotechnol. Rep..

[11] Hasanuzzaman M, Nahar K, Fujita M, Ahmad P, Azooz MM, Prasad MNV (2013). Plant response to salt stress and role of exogenous protectants to mitigate salt-induced damages. Ecophysiology and Responses of Plants under Salt Stress.

[12] Nassar RM (2020). Physiological and anatomical mechanisms in wheat to cope with salt stress induced by seawater. Plants.

[13] Naz T (2021). Foliar application of potassium mitigates salinity stress conditions in spinach (*Spinacia oleracea* L.) through reducing nacl toxicity and enhancing the activity of antioxidant enzymes. Horticulturae.

[14] Peng K (2008). Manganese uptake and interactions with cadmium in the hyperaccumulator-*Phytolacca Americana* L.. J. Hazard. Mater..

[15] Zafar S (2021). Deciphering physio-biochemical characteristics of ZnSO_4_ primed wheat (Triticum aestivum L.) plants grown under salt stress.. Pak. J. Bot.

[16] Maas EV, Grieve CM (1990). Spike and leaf development of sal-stressed wheat. Crop Sci..

[17] Al Sabagh A (2021). Salinity stress in wheat (*Triticum aestivum* L.) in the changing climate: Adaptation and management strategies. Front. Agron..

[18] Smith SE, Read DJ (2010). Mycorrhizal Symbiosis.

[19] Srivastava P, Saxena B, Giri B, Varma A, Prasad R, Tuteja N (2017). Arbuscular mycorrhizal fungi: green approach/technology for sustainable agriculture and environment. Mycorrhiza-Nutrient Uptake, Biocontrol, Ecorestoration.

[20] Ryan MH, Graham JH (2018). Little evidence that farmers should consider abundance or diversity of arbuscular mycorrhizal fungi when managing crops. New Phytol..

[21] Jajoo A, Mathur S (2021). Role of arbuscular mycorrhizal fungi as an underground savior for protecting plants from abiotic stresses. Physiol. Mol. Biol. Plants.

[22] Thirkell TJ, Charters MD, Elliott AJ, Sait SM, Field KJ (2017). Are mycorrhizal fungi our sustainable saviours? Considerations for achieving food security. J. Ecol..

[23] Begum N (2019). Role of arbuscular mycorrhizal fungi in plant growth regulation: Implications in abiotic stress tolerance. Front. Plant Sci..

[24] Klinsukon C, Lumyong S, Kuyper TW, Boonlue S (2021). Colonization by Arbuscular mycorrhizal fungi improves salinity tolerance of eucalyptus (*Eucalyptus camaldulensis*) seedlings. Sci. Rep..

[25] Talaat NB, Shawky BT (2014). Protective effects of arbuscular mycorrhizal fungi on wheat (*Triticum aestivum* L.) plants exposed to salinity. Environ. Exp. Bot..

[26] Hajiboland R, Aliasgharzadeh N, Laiegh SF, Poschenrieder C (2010). Colonization with arbuscular mycorrhizal fungi improves salinity tolerance of tomato (*Solanum lycopersicum* L.) plants. Plant Soil.

[27] Talaat NB, Shawky BT (2011). Influence of arbuscular mycorrhizae on yield, nutrients, organic solutes, and antioxidant enzymes of two wheat cultivars under salt stress. J. Plant Nutr. Soil Sci..

[28] Ministry of Food, Agriculture & Livestock (2007). Agricultural Statistics of Pakistan.

[29] Kanwal S (2017). Application of biochar in mitigation of negative effects of salinity stress in wheat (*Triticum aestivum* L.). J. Plant Nutr..

[30] Von Braun, J. & Bos, M. S. *The changing economics and politics of rice: implications for food security, globalization, and environmental sustainability*. *IRRI reports* (2005).

[31] Raza, A. Grain and field annual 2012 grain report. *Global Agricultural Information Network. USDA Foreign Agricultural Services* 1–3 at (2012).

[32] Badridze G, Weidner A, Asch F, Börner A (2009). Variation in salt tolerance within a Georgian wheat germplasm collection. Genet. Resour. Crop Evol..

[33] Munns R, James RA, Läuchli A (2006). Approaches to increasing the salt tolerance of wheat and other cereals. J. Exp. Bot..

[34] Rashid A, Saleem Q, Nazir A, Kazım HS (2003). Yield potential and stability of nine wheat varieties under water stress conditions. Int. J. Agric. Biol..

[35] Jackson ML (2005). Soil Chemical Analysis: Advanced Course.

[36] Olsen SR, Sommers LE, Page AL (1982). Phosphorus. Method of Soil Analysis, Agron. No. 9, Part 2: Chemical and Microbiological Properties.

[37] Nelson DW, Sommers LE, Page AL (2015). Total carbon, organic carbon, and organic matter. Methods of Soil Analysis, Part 2.

[38] Alam MZ, Carpenter-Boggs L, Hoque MA, Ahammed GJ (2020). Effect of soil amendments on antioxidant activity and photosynthetic pigments in pea crops grown in arsenic contaminated soil. Heliyon.

[39] Jagatheeswari D, Ranganathan P (2012). Influence of Mercuric Chloride on seeds germination, seedling growth and biochemical analysis of green gram (*Vigna radiata* (L.) Wilczek. Var. Vamban-3). Int. J. Pharm. Biol. Arch..

[40] Kumar S (2021). Effect of salt stress on growth, physiological parameters, and ionic concentration of water dropwort (*Oenanthe javanica*) cultivars. Front. Plant Sci..

[41] Hariadi Y, Marandon K, Tian Y, Jacobsen SE, Shabala S (2011). Ionic and osmotic relations in quinoa (*Chenopodium quinoa* Willd.) plants grown at various salinity levels. J. Exp. Bot..

[42] Mazhar R, Ilyas N, Saeed M, Bibi F, Batool N (2016). Biocontrol and salinity tolerance potential of *Azospirillum lipoferum* and its inoculation effect in wheat crop. Int. J. Agric. Biol..

[43] Arnon DI (1949). Copper enzymes in isolated chloroplasts. Polyphenoloxidase in *Beta vulgaris*. Plant Physiol..

[44] Khan MY, Haque MM, Molla AH, Rahman MM, Alam MZ (2017). Antioxidant compounds and minerals in tomatoes by Trichoderma-enriched biofertilizer and their relationship with the soil environments. J. Integr. Agric..

[45] Melandri G (2021). Drought tolerance in selected aerobic and upland rice varieties is driven by different metabolic and antioxidative responses. Planta.

[46] Beauchamp CO, Fridovich I (1973). Isozymes of superoxide dismutase from wheat germ. Biochim. Biophys. Acta Protein Struct..

[47] Maehly A, Chance B (1954). Catalases and peroxidases. Methods Biochem. Anal..

[48] Aebi HE (1983). Catalase. Methods of Enzymatic Analysis.

[49] Velikova V, Yordanov I, Edreva A (2000). Oxidative stress and some antioxidant systems in acid rain-treated bean plants. Plant Sci..

[50] Rao KM, Sresty TVS (2000). Antioxidative parameters in the seedlings of pigeonpea (*Cajanus cajan* (L.) Millspaugh) in response to Zn and Ni stresses. Plant Sci..

[51] Ahmad P (2016). Calcium and potassium supplementation enhanced growth, osmolyte secondary metabolite production, and enzymatic antioxidant machinery in cadmium-exposed chickpea (*Cicer arietinum* L.). Front. Plant Sci..

[52] Talaat NB, Shawky BT (2013). 24-Epibrassinolide alleviates salt-induced inhibition of productivity by increasing nutrients and compatible solutes accumulation and enhancing antioxidant system in wheat (*Triticum aestivum* L.). Acta Physiol. Plant..

[53] Elgharably A, Nafady NA (2021). Inoculation with Arbuscular mycorrhizae, *Penicillium funiculosum* and *Fusarium oxysporum* enhanced wheat growth and nutrient uptake in the saline soil. Rhizosphere.

[54] Ndiate NI (2021). Co-application of biochar and arbuscular mycorrhizal fungi improves salinity tolerance, growth and lipid metabolism of maize (*Zea mays* L.) in an alkaline soil. Plants.

[55] Abdel Latef A (2010). Changes of antioxidative enzymes in salinity tolerance among different wheat cultivars. Cereal Res. Commun..

[56] Shokri S, Maadi B (2009). Effects of arbuscular mycorrhizal fungus on the mineral nutrition and yield of *Trifolium alexandrinum* plants under salinity stress. J. Agron..

[57] Kaya C (2009). The influence of arbuscular mycorrhizal colonisation on key growth parameters and fruit yield of pepper plants grown at high salinity. Sci. Hortic. (Amsterdam).

[58] Ruiz-Lozano JM, Azc’on R (2000). Symbiotic efficiency and infectivity of an autochthonous arbuscular mycorrhizal *Glomus* sp. from saline soils and *Glomus deserticola* under salinity. Mycorrhiza.

[59] Latef AAHA, Chaoxing H (2011). Arbuscular mycorrhizal influence on growth, photosynthetic pigments, osmotic adjustment and oxidative stress in tomato plants subjected to low temperature stress. Acta Physiol. Plant..

[60] Murkute AA, Sharma S, Singh SK (2006). Studies on salt stress tolerance of citrus rootstock genotypes with arbuscular mycorrhizal fungi. Hortic. Sci..

[61] Giri B, Kapoor R, Mukerji KG (2003). Influence of arbuscular mycorrhizal fungi and salinity on growth, biomass, and mineral nutrition of *Acacia auriculiformis*. Biol. Fertil. Soils.

[62] Zuccarini P (2007). Mycorrhizal infection ameliorates chlorophyll content and nutrient uptake of lettuce exposed to saline irrigation. Plant Soil Environ..

[63] Berman HM (2000). The protein data bank. Nucleic Acids Res..

[64] Davis RF, Earl HJ, Timper P (2014). Effect of simultaneous water deficit stress and meloidogyne incognita infection on cotton yield and fiber quality. J. Nematol..

[65] Liang BB (2021). Arbuscular mycorrhizal fungi can ameliorate salt stress in *Elaeagnus angustifolia* by improving leaf photosynthetic function and ultrastructure. Plant Biol..

[66] Zhao H (2019). Effects of salt stress on chlorophyll fluorescence and the antioxidant system in *Ginkgo biloba* L. seedlings. HortScience.

[67] Evelin H, Kapoor R (2014). Arbuscular mycorrhizal symbiosis modulates antioxidant response in salt-stressed *Trigonella foenum-graecum* plants. Mycorrhiza.

[68] Evelin H, Giri B, Kapoor R (2013). Ultrastructural evidence for AMF mediated salt stress mitigation in *Trigonella foenum-graecum*. Mycorrhiza.

[69] Singh RP, Prasad PVV, Sunita K, Giri SN, Reddy KR (2007). Influence of high temperature and breeding for heat tolerance in cotton: A review. Adv. Agron..

[70] Ruiz-Lozano JM, Azcón R (2000). Symbiotic efficiency and infectivity of an autochthonous arbuscular mycorrhizal *Glomus* sp. from saline soils and *Glomus deserticola* under salinity. Mycorrhiza.

[71] Mohammad MJ, Malkawi HI, Shibli R (2003). Effects of arbuscular mycorrhizal fungi and phosphorus fertilization on growth and nutrient uptake of barley grown on soils with different levels of salts. J. Plant Nutr..

[72] Al-Karaki GN, Hammad R, Rusan M (2001). Response of two tomato cultivars differing in salt tolerance to inoculation with mycorrhizal fungi under salt stress. Mycorrhiza.

