# Supplementary material for: Uncovering the impact of AM fungi on wheat nutrient uptake, ion homeostasis, oxidative stress, and antioxidant defense under salinity stress
Source: Sci Rep. 2023 May 22;13:8249. doi: 10.1038/s41598-023-35148-x (PMC10202960; doi:10.1038/s41598-023-35148-x)
Supplement: Supplementary file 1 — Supplementary Figures. [file 41598_2023_35148_MOESM1_ESM.docx]

**Uncovering the Impact of AM Fungi on Wheat Nutrient Uptake, Ion Homeostasis, Oxidative Stress, and Antioxidant Defense under Salinity Stress**

Shoucheng Huang^1^*, Sidra Gill^2^, Musarrat Ramzan^2^, Muhammad Zaheer Ahmad^3^, Subhan Danish^4^, Ping Huang^5^, Sami Al Obaid^6^, Sulaiman Ali Alharbi^6^

^1^College of Life and Health Science, Anhui Science and Technology University, Fengyang 233100,China; [Huangsc@ahstu.edu.cn](mailto:Huangsc@ahstu.edu.cn)

^2^Department of Botany, Faculty of Chemical & Biological Sciences, The Islamia University of Bahawalpur, Pakistan; [sidragill68f@gmail.com](mailto:sidragill68f@gmail.com); [musarrat.ramzan@iub.edu.pk](mailto:musarrat.ramzan@iub.edu.pk)

^3^Dr. M. Ajmal Khan, Insititute of Sustainable Halophytes Utilization, Univerisity of Karachi, Pakistan; [zaheerahmad@Ku.edu.pk](mailto:zaheerahmad@Ku.edu.pk)

^4^Department of Soil Science, Faculty of Agricultural Sciences and Technology, Bahauddin Zakariya University, Multan, Punjab, Pakistan; [sd96850@gmail.com](mailto:sd96850@gmail.com)

^5^College of Chemistry and Materials Engineering, Anhui Science and Technology University, Bengbu 233000,China; [Huangp@ahstu.edu.cn](mailto:Huangp@ahstu.edu.cn)

^6^Department of Botany and Microbiology, College of Science, King Saud University, PO Box -2455, Riyadh-11451, Saudi Arabia; [saalobaid@ksu.edu.sa](mailto:saalobaid@ksu.edu.sa); [sharbi@ksu.edu.sa](mailto:sharbi@ksu.edu.sa)

Corresponding author: [Huangsc@ahstu.edu.cn](mailto:Huangsc@ahstu.edu.cn); [musarrat.ramzan@iub.edu.pk](mailto:musarrat.ramzan@iub.edu.pk); [sd96850@gmail.com](mailto:sd96850@gmail.com);

**Figure S1.** Principal component analysis for studied attributes keeping AMF as main observation

**Figure S2.** Principal component analysis for studied attributes keeping salinity as main observation
